# The Book World of Medicine and Science

**Published:** 1902-08-02

**Authors:** 


### THE BOOK WORLD OF MEDICINE AND SCIENCE.

THE NORDRACH TREATMENT OF CONSUMPTION IN THIS COUNTRY. By JAMES ARTHUR GIBSON. (London: Sampson Low, Marston, and Co. 1901. Pp. 163. Price 3s. 6d. net).

THE author of this book is a gentleman of scientific training who, having contracted pulmonary phthisis, spent some time at Nordrach under the care of Dr. Walther, and happily derived permanent benefit from his stay. Since his return to England he has enthusiastically preached the advantages of the Nordrach methods, and, indeed, the present volume is a revised reprint of articles which have already appeared in a popular monthly review. Enthusiasm is valuable, no doubt, and it is in great measure the result of enthusiastic advocacy that so much attention is now paid, in the treatment of pulmonary phthisis, to the observation of the strict rules of hygiene. But this book should, we think, receive more critical consideration than is sometimes given to the writings of enthusiasts, especially as it evidently aims at a popular vogue. Briefly, Mr. Gibson's case, as stated by himself, is this:—He broke down in health in 1895, at the age of 28, after 18 months or so of illness, during which period he had not recognised the cause of his excessive languor and weakness. He was then examined by eminent practitioners, who diagnosed phthisis. He was ordered to the country, where he gained weight. Subsequently, on the advice of a friend, he visited Nordrach, and left that place in three months "cured." Since then Mr. Gibson has continued well. Very possibly if Mr. Gibson had observed the ordinary rules of hygiene during his 18 months of excessive languor he would not have broken down, and if he had not gained weight during his enforced stay in Ireland he might not have derived much benefit from his stay at Nordrach. The point we wish to make is that the disease was evidently not progressive when he went to Nordrach, and that, as Mr. Gibson himself says, since his return he has taken "more care to rest and nourishment" and has avoided "the things such as impure air," which brought about the breakdown. Yet Mr. Gibson, after three months' stay at Nordrach, has the confidence to italicise this dogma:—"That climate certainly has nothing to do with either the cause or the cure of consumption," and to make the preposterous statement that "not a single life need be lost through consumption, if only the proper means of grappling with it are employed in time!" Not content with gibing at the profession to which he owes his life, Mr. Gibson indulges in wanton sarcasm at the expense of our great Consumption Hospitals, to the beneficent work of which, as Koch recently pointed out, is in great measure to be ascribed the diminution, that has for years been going on, of our phthisis mortality. Mr. Gibson means well, and is without doubt sincere. But his book is a medley of ill-digested information and unevenly balanced argument, interspersed with sarcasm at the expense of those who laboured for years before Mr. Gibson went on his three months' visit to Nordrach.

THE WAY OF ESCAPE. By GRAHAM TRAVERS (Margaret Todd, M.D.). (London: William Blackwood and Sons. 1902. Price 6s.)

PREVIOUS works by Graham Travers have shown that she is able to tell a tale in a convincing fashion and that she can make a story serve a purpose without detracting from its interest, and in "The Way of Escape" we have another example of the power of her pen and of her careful study of human nature. The tale is admirably told, the interest is well maintained, the *finale* is tragic and dramatic, and, for those who do not care to seek the moral, that is probably enough. But behind it all one sees how different are the consequences to the man and to the woman of certain errors, the fault of which may lie equally between them, and this one may take as the teaching of the book. The episode being over the man turns over the page, definitely and for good; he enters on his life's work, which has nothing to do with love or its results. But what was to the man an episode is to the woman an influence which cannot be lived down or forgotten, for though she may seem to turn the page, it is always liable to fly open and display the past to herself and to the world. The story here is of a girl without religious training, but with great intelligence and nobility of mind, and a young man, the best and final product of those educational advantages and religious and social prejudices which abound in Edinburgh. They err and then they separate. He becomes a successful physician, who, although tied to a highly religious termagant of a wife, is wealthy, prosperous, and highly thought of, while she spends her life trying to escape the social penalties which follow such a fault. She nearly achieves happiness, but draws back rather than risk confession or exposure; finally she devotes herself to working for the good of others, but the old story persists in cropping up, and when she dies, death seems rather a way of escape than a terror to avoid. If we are to indulge in criticism of such a book, we should suggest that perhaps too much is left to the imagination. We are left to judge of the extent of Vera's error by the intensity of its effect upon her life, and although the abiding nature and all-powerful influence of her one fault is depicted with striking force, we are all the time left in considerable doubt as to what is the motive of much that she does. At one moment it seems as if it were a desire to be level with the man who has deserted her, at another a determination not to be beaten by the world, at another even an ordinary common dread of being found out. All this detracts from the unity of the picture; but perhaps, as after all heroines are but women, it is none the less true to life.

"YEAR-BOOK OF THE SCIENTIFIC AND LEARNED SOCIETIES OF GREAT BRITAIN AND IRELAND." Compiled from Official Sources. (London: Charles Griffin and Co. 1901).

THIS is a work the compilation of which must have entailed a great deal of labour, giving as it does a list of the papers read from January 1900 to June 1901 before the various societies classified under 14 different departments of research, with the names of their authors and of the officials of the various societies.

